# Kochiae Fructus, the Fruit of Common Potherb *Kochia scoparia* (L.) Schrad: A Review on Phytochemistry, Pharmacology, Toxicology, Quality Control, and Pharmacokinetics

**DOI:** 10.1155/2021/5382684

**Published:** 2021-01-31

**Authors:** Wei Zou, Zhong Tang, Yao Long, Zuoqi Xiao, Bo Ouyang, Menghua Liu

**Affiliations:** ^1^NHC Key Laboratory of Birth Defects Research, Prevention and Treatment, Hunan Provincial Maternal and Child HealthCare Hospital, Changsha 410008, China; ^2^Breast Disease Department, The First Hospital of Hunan University of Chinese Medicine, Changsha 410007, China; ^3^Guangdong Provincial Key Laboratory of New Drug Screening, School of Pharmaceutical Sciences, Southern Medical University, Guangzhou 510515, China

## Abstract

Kochiae Fructus (KF) is the fruit of an annual potherb *Kochia scoparia* (Linn.) Schrad and has been traditionally used for the treatment of diseases in the skin, eyes, and urinary tract for thousands of years in China. Recent studies have showed its anti-inflammatory, antifungal, antiallergic, and antipruritogenic effects to clarify the mechanisms of these actions. Meanwhile, its other effects, such as anticancer, hypoglycemic, and hepatoprotective effects, also have been reported. The achievement of these therapeutic effects is contributed by its chemical constituents. A total of 153 compounds have been identified in KF, mainly including triterpenoids, flavonoids, carbohydrates, amino acids, organic acids, and essential oils. Momordin Ic is the representative triterpene glycoside compound, which is used as a phytochemical marker for the quality control of Kochiae Fructus. The research on toxicity is insufficient, and only one article reported that the LD_50_ was 7.15 ± 0.03 g/kg for water extract of KF after oral administration in KM mice. In addition, the pharmacokinetic study was carried out on momordin Ic with linear pharmacokinetic characteristics. Above all, this review provides comprehensive information about Kochiae Fructus and may provide the theoretic foundation of its clinical application and further development.

## 1. Introduction

**Figure 1:**
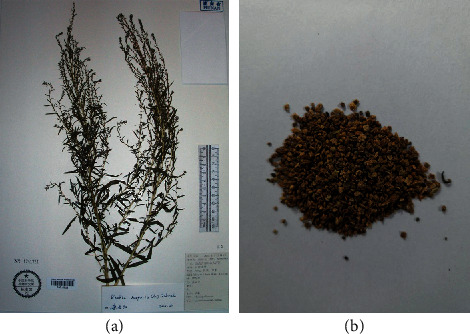
(a) The potherb of *Kochia scoparia* (L.) Schrad (http://www.cvh.ac.cn/spms/detail.php?id=f27a89aa) and (b) its fruit Kochiae Fructus.

*Kochia scoparia* (Linn.) Schrad (shown in Figure 1(a)), also called *Bassia scoparia* (L.) A.J. Scott, is a large annual potherb in the family Chenopodiaceae widely distributed in Europe and Asia and naturalized in Africa, Australia, and North and South America [1]. Kochia Fructus (KF, shown in Figure 1(b)) is the fruit of *Kochia scoparia*, which is a spheroidal pentagram with a diameter of 1 to 3 mm [2]. It was first recorded in “Shennong Ben Cao Jing” as a “top grade” medicinal material. Up to now, KF has been used in traditional Chinese and Japanese medicine more than 2000 years for the treatment of diseases of the skin, eyes, and urinary tract [3]. With the deepening and development of pharmacology research, it has attracted attention particularly because of its antibacterial, anti-inflammatory, antiallergic, antigastric mucosal damage, hypoglycemic, and immunity enhancing effects [4]. Recently, researchers demonstrated that KF mainly contains terpenoids, flavonoids, essential oils, trace elements, and other ingredients. Although there were many researches on the chemical constituents, pharmacological activities of KF, a systematic and updated review is unavailable. Therefore, the aim of this review is to extensively summarize the phytochemistry, pharmacology, quality control, toxicology, and pharmacokinetics of KF, as well as providing novel insights for clinical uses and further researches.

## 2. Phytochemistry

**Figure 2:**
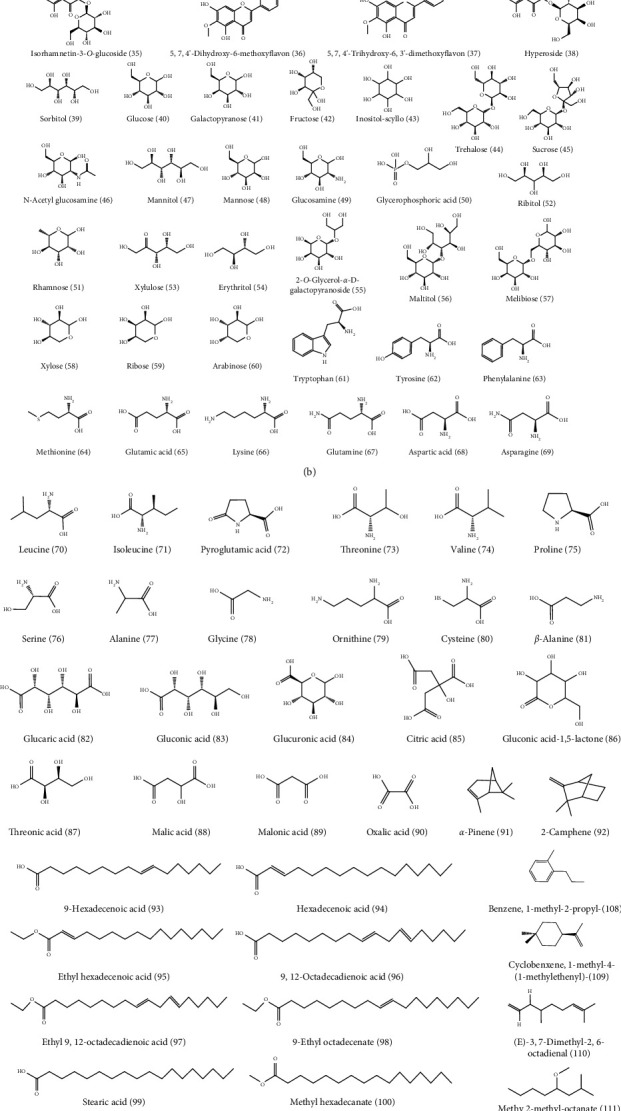
Chemical structures of substances within Kochiae Fructus.

With the advancement of analysis technologies such as liquid chromatograph-mass spectrometer (LC-MS), nuclear magnetic resonance-mass spectrometer (NMR-MS), and gas chromatography-mass spectrometer (GC-MS), identification of various components in traditional Chinese medicine has been simplified. To date, 153 compounds within KF, including 25 triterpenoids, 13 flavonoids, 22 carbohydrates (primarily mono- and disaccharides), 21 amino acids, 9 organic acid, 49 essential oils, and 14 heterocyclics, have been identified (Table 1 and Figure 2). Most of investigations indicated that triterpenoids are the main active ingredient within KF. They were characterized with tetracyclic or pentacyclic rings by the polymerization of isoprene. Among them, momordin Ic is a representative triterpenoid saponin with anti-inflammatory effect [5]. Flavonoids were another major component within KF [12]. Most of them are derivatives of flavonol aglycones including quercetin and isorhamnetin. The carbohydrates of flavonoid glycosides are glucopyranose, rhamnose, and galactose. Besides, other flavonoids such as 5,7,4′-trihydroxy-6,3′-dimethoxyflavone and 5,7,4′-dihydroxy-6-methoxyflavone were characterized by LC-MS [6]. KF contains many kinds of amino acid, and current research suggests that certain functional amino acids can play a pharmacological role through the gut-microbiome-immune axis [17]. The essential oil within KF is high fatty acid ester. Yang et al. used a supercritical CO_2_ extraction method combined with a gas chromatography-mass spectrometer (GC-MS) method to qualify these essential oil components [14]. Eighteen compounds were isolated and identified, most of which were fatty acid esters and aromatic compounds. The level of the higher fatty acid ester is high, and the relative amount of 9,12-octadecadienoic acid is the highest in oil, followed by 9-octadecenoic acid. Wen et al. used the GC-MS method to qualitatively analyze the essential oil within KF [15]. Compared with the standard mass spectrum, 36 components were identified. Among them, the relative level of high fatty acid esters is the highest, and the amount of terpenoids is small.

## 3. Pharmacology

Traditionally, according to records of “Shennong Ben Cao Jing,” KF was used with the therapeutic effects of diuresis and benefiting pneuma. Compendium of Materia Medica described that the KF could be used in the treatment of red eyes, hemntodiarrhoea, pregnancy combined with gonorrhea, and urinary stoppage [18]. Many other books also depicted the traditional use of KF, which are summarized in Table 2. Modern investigations have proved that KF has anti-inflammatory, hypoglycemic, anticancer, antifungal, antipruritogenic, and antinociceptive effects, as well as antiallergic, antiedema, and hepatoprotective activities. We have enlisted an overview of the pharmacological studies in the following sections (Table 3).

### 3.1. Anti-Inflammatory Effect

Pharmacological studies showed that anti-inflammatory is a very significant pharmacological activity of KF. In six different animal models (the ddY mice in an acetic acid-induced vascular permeability, the ddY mice in a carrageenin-induced edema, the ddY mice in a compound 48/80-induced edema, the ddY mice in a chemical mediator-induced edema, the ddY mice in an arachidonic acid-induced edema, a picryl chloride-induced ear inflammatory model in ICR mice), the 70% alcohol extract of KF has been proved with obvious inhibition effect on the development of inflammation [19, 20]. The methanol extract of KF was used as a candidate drug for the treatment of inflammatory skin diseases due to its eutherapeutic effect on 1-fluoro-2,4-dinitrofluorobenzene-induced contact dermatitis mice model. The mechanism might be involved in inhibiting the skewing reaction of T helper cell type 1 [22]. The total flavonoids of the KF have shown an anti-inflammatory effect on the dinitrochlorobenzene-induced allergic contact dermatitis rats, and the most likely mechanism of this action involves regulating pERK1/2/TLR4-NF-*κ*B pathway activation [42]. The anti-inflammatory effect of KF was coincident with its traditional use for inflammations in vagina and skin.

Three triterpenoid saponins, namely, 20-hydroxyecdysone, momordin Ic, and oleanolic acid from KF have also been investigated on LPS-stimulated murine macrophage RAW 264.7 cell line. 20-Hydroxyecdysone performed significant inhibitory action on prostaglandin E_2_ (PGE_2_) generation at the dose of 12.5 *μ*M, while momordin Ic and oleanolic acid showed the anti-inflammatory effect at the dose of 6.25 *μ*M. In addition, momordin Ic significantly reduced productions of tumor necrosis factor-alpha (TNF-*α*) and interleukin-6 (IL-6) at the concentration of 12.5 *μ*M [5].

### 3.2. Hypoglycemic Effect

KF has shown a potential hypoglycemic effect. Dai et al. illustrated that *n*-butanol fraction of KF could markedly inhibit gastric emptying in normal mice and could more potently inhibit gastric emptying in hyperglycemic and hypoglycemic mice at the dose of 25 mg/kg. The hypoglycemic mechanism is probably related to transportation and transformation of sugar in the digestive tract and absorption of glucose via the membrane of the small intestine [23]. Subsequently, a research on the function of small intestine was implemented and it found that the *n*-butanol fraction with a dose of 50 mg/kg could improve the propulsive function of small intestine, and the mechanism of this action probably involves cholinergic nerve and nitric oxide [24].

Matsuda et al. found that momordin Ic inhibited gastric emptying in rats and inhibited glucose uptake in the small intestine *in vitro*, which contributed to the hypoglycemic action of momordin Ic [25]. Further study showed that momordin Ic inhibits gastric emptying in normal mice, hyperglycemic (including diabetic) and hypoglycemic mice, nonnutrient meal-loaded mice, and nutrient meal-loaded mice [3]. When gastric emptying is slow, the postprandial absorption of food will prolong. Hence, the inhibition of gastric emptying induced by momordin Ic may be useful for the prevention and treatment of diabetes and the morbid obesity with accelerated gastric emptying.

### 3.3. Anticancer Effect

#### 3.3.1. Antiliver Cancer Effect

**Figure 3:**
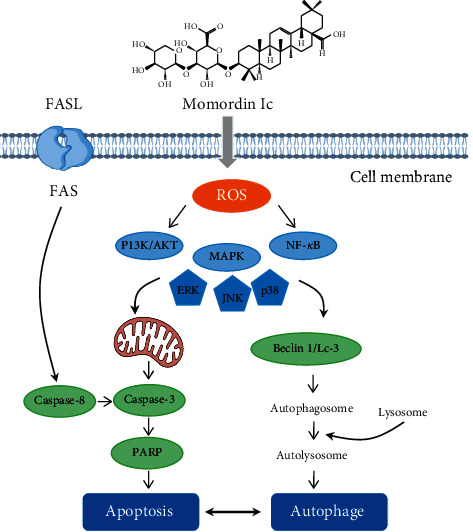
The signal pathways of antiliver cancer effect of momordin Ic.

Momordin Ic was the main triterpenoid saponins within KF and has showed an antiliver cancer effect. Wang et al. have carried out a series of researches and found that HepG2 cells were sensitive to the cytotoxic effect of momordin Ic. Momordin Ic could induce apoptosis through oxidative stress-regulated mitochondrial dysfunction involving MAPK and PI3K-mediated iNOS and HO-1 pathways [26]. Based on these results, Wang et al. investigated the MAPK and PI3K pathways and their downstream proteins, such as PPARg and COX-2. Then, they provided the evidence that momordin Ic-induced HepG2 cell apoptosis was associated with PI3K and MAPK pathway-mediated PPARg activation [28]. In addition, Mi et al. showed that the underlying mechanisms of the cross-talk between apoptosis and autophagy involved ROS-related PI3K/Akt, MAPK, and NF-*κ*B signaling pathways, and momordin Ic simultaneously induced apoptosis and autophagy by activating these intersecting signaling pathways [27]. The summarized signal pathway is presented in Figure 3. On the contrary, momordin Ic showed a good anti-invasive activity by altering E-cadherin, VCAM-1, ICAM-1, and MMP-9, and the underlying mechanism involved PPAR*γ* activation and COX-2 inhibition [30].

#### 3.3.2. Antiprostate Cancer Effect

The MeOH extract of KF has shown inhibition effects on human umbilical vein endothelial cell angiogenesis and human prostate cancer cell proliferation [31]. As a member of the de-SUMOylation protease family, SUMO-specific protease 1 (SENP1) is elevated in prostate cancer (PCa) cells and is involved in PCa pathogenesis [43–46]. Momordin Ic as a novel SENP1 inhibitor could inhibit proliferation of prostate cancer cells *in vitro* and *in vivo* by inducing cell cycle arrest and apoptosis [32]. The possible mechanism is that momordin Ic could increase the sub-G1 phase cell population, increase numbers of annexin-V positive cells, increase active caspase-3, caspase-8, and PARP1 cleavage, and reduce cyclin B and CDK1 levels. Thus, it was considered that SENP1 may play an important role in momordin Ic-induced cell death in prostate cancer cells, even though the downstream effectors of SENP1 that mediate momordin Ic-induced apoptosis are currently unknown.

### 3.4. Antifungal Effect

*In vitro*, the water extract of KF showed a strong inhibition on common dermatophytes. Its minimum inhibitory concentration (MIC) on *Trichophyton mentagrophytes* was 3.12% and on *Trichophyton rubrum*, *Microsporum canis*, *Trichophyton violaceum*, and *Trichophyton schoenleinii* were 0.78% [33]. Wu et al. tested six KF extracts against *Fusarium graminearum*, *Fusarium oxysporum*, *Monilia cinerea*, *Physalos porapiricola*, *Alternaria alternata*, and *Valsa mali.* As a result, the water extract had the strongest inhibition effect on all six plant pathogenic bacteria with antifungal activities of more than 74.34%, and the water, petroleum ether, chloroform, ethylacetate, and methanol extracts showed stronger antifungal activities against *Monilia cinerea* and *Valsa mali* than the others [34]. In addition, the saponin extract, flavone extract I (40% alcohol eluent), flavone extract II (80% alcohol eluent), and lipid extract from KF were tested against *Microsporum ferrugineum, Microsporum gypseum, Trichophyton schoenleini, Trichophyton mentagrophytes, Trichophyton violaceum, Trichophyton rubrum, Epidermophyton floccosum, Aspergillus fumigatus, Candida albicans,* and *Cryptococcus neoformans*. The lipid extract showed a good antifungal effect on *Microsporum ferrugineum*, *Microsporum gypseum, Trichophyton mentagrophytes,* and *Trichophyton rubrum*; the saponin extract gave inhibition effects on *Microsporum ferrugineum, Microsporum gypseum, Trichophyton schoenleini, Trichophyton mentagrophytes,* and *Trichophyton rubrum*; the flavone extract I (40% alcohol eluent) exerted inhibition effects on *Microsporum ferrugineum, Trichophyton rubrum,* and *Epidermophyton floccosum*, whereas the flavone extract II (80% alcohol eluent) play an inhibitory role on *Microsporum ferrugineum, Microsporum gypseum,* and *Trichophyton rubrum*. Unfortunately, the MIC was not mentioned in this article [35]. Above all, this effect of KF supports its traditional use in gynecological infection.

### 3.5. Antipruritogenic Effect

The 70% ethanol extract (200 mg/kg) and methanol extract of KF (500 mg/kg) have been proved to inhibit the scratching behavior on a compound 48/80-induced pruritogenic model in male ddY mice [36]. Momordin Ic isolated from KF also exhibited an inhibition effect at a dose of 50 mg/kg. Meanwhile, in an itching guinea pig model and an itching mice model, the water extract of KF at the concentration of 0.15 g/mL could significantly decrease the number of itching and total time of itching within 30 minutes, indicating that KF could be used as an antipruritogenic agent [33]. These results agree with the traditional use of KF for itch.

### 3.6. Others

The inhibition effect of hypersensitivity of 70% ethanol and total saponin extracts from KF has been tested on DTH models upon challenge with SRBC or PC. As a result, the 70% ethanol extract produced a concentration-dependent reduction on immediate and delayed-type hypersensitivity, while total saponins extract showed an inhibitory tendency at 200 mg/kg [20]. This effect might have a close relationship with its stabilization of mast cell membrane, reduction of release of anaphylactic mediators, and anti-inflammatory activities [20]. The water extract of KF at the dose of 20 mg/g could reverse the imbalance of Th1/Th2 cell in rat model with dilated cardiomyopathy, which reduced the damage of immune response to myocardium and protected the heart function. Momordin Ic gave a protective effect on gastric mucosal lesions [37]. Kim et al. discovered that momordin Ic has a hepatoprotective effect against CCl_4_-induced liver damage because it could enhance the hepatic antioxidant defense system [38]. Meanwhile, as an AP-1 inhibitor, momordin Ic could downregulate NF-*κ*B activation as well as AP-1 activation, which plays a key role in osteoclast differentiation, by inhibiting I*κ*B degradation and c-Fos expression, respectively. Therefore, momordin Ic has high potential to be a good candidate for controlling bone disorders in the future [40]. In addition, the extracts of KF, including total flavonoid, saponin, and phenolic, were proved to have a good antioxidant activity by measuring free radical scavenging activities with ABTS, DPPH, or FARP assay [47–50]. Scavenging free radicals play a key role in aging and inflammation [51]. Therefore, its antioxidant effects need to be further studied on these aspects.

The studies on toxicity of KF are scarce. Although KF is almost nontoxic in traditional use, the animal death was found when a large dosage is used. According to a toxicological study, when KM mice were orally administrated with water extract of KF in a dose range from 4.5 mg/kg to 9.4 mg/kg, the median lethal dose (LD_50_) was 7.15 ± 0.03 g/kg [35]. Therefore, the toxic and side effects of KF should be paid more attention in its clinical application.

## 4. Quality Control

The quality of herbal medicines is the key for its clinical efficacy and safety, and hence, establishing a quality control system is the premise of its clinical application. The quality control of KF was focused on quantitative analysis of components by a series of analytical methods, such as ultraviolet-visible detector (UV), gas chromatography-mass spectrometry (GC-MS), and high-performance liquid chromatography-evaporative light scattering detector (HPLC-ELSD). Since the main component of KF is triterpenoid saponin, whose UV absorption is poor, researchers incline to use ELSD. Xia et al. developed a method to determine the content of momordin Ic in KF and the content of momordin Ic was 0.83%–0.21% in four kinds of marketed KF [52]. Moreover, they used HPLC-ELSD and colorimetric methods to determine the content of momordin Ic and total saponins, respectively, in KF at different collecting times or from eleven places in China [53, 54]. Except for saponin, there also established an HPLC method with good stability and reproducibility to simultaneously determine the content of rutin and quercetin in KF [55]. Currently, the fingerprint derived from HPLC has been an acknowledged method to control the quality of traditional Chinese medicine and botanical medicine. An HPLC fingerprint method was applied to 10 batches of KF purchased from Shandong, China. According to the cluster analysis, 19 common peaks of fingerprint were found, and 10 batches could be divided into 3 groups related to their origins [56]. The results showed that this method could differentiate samples from different geographical origins or processing methods. Nevertheless, there were few compounds quantified as mark compounds for the quality control of KF, and it is in urgent need of a comprehensive quantification method to further ensure the quality control.

## 5. Pharmacokinetics

Momordin Ic is a representative pharmacologically active ingredient and quality control marker of KF, and its pharmacokinetic property has attracted attentions. Yan et al. developed and validated a highly selective and sensitive method based on ultraperformance liquid chromatography-tandem mass spectrometry (UPLC-MS/MS) for routine analysis of momordin Ic in rat plasma. After intravenous administration of momordin Ic at 0.52, 1.56, and 4.67 mg/kg in rats, the AUC_last_ (area under the concentration-time curve from time 0 to *t* hours postodose) values were 1864.17 ± 431.01, 5466.00 ± 889.86, and 16890.45 ± 3028.64 ng h/mL, respectively, which was consistent with linear pharmacokinetic characteristics. The elimination half-life (*t*_1/2_) values were 1.22 ± 0.39, 1.14 ± 0.10, and 1.83 ± 0.39 h, respectively [4]. However, there are few studies on the pharmacokinetic of other substances within KF and on the interaction between substances during their ADME *in vivo*.

## 6. Conclusion and Perspectives

Recent pharmacology studies showed anti-inflammatory, antifungal, antiallergic, and antipruritogenic effects of KF, which supports the traditional clinical applications including the treatment of diseases in the skin, eye, and urinary tract in China, Korea, and Japan. Interestingly, the anticancer, hypoglycemic, and hepatoprotective effects of KF were also tested. Besides, the potential mechanisms of some effects were also elucidated. However, there are few toxicology studies on KF, which may be necessary for its better application as a medicine or a food. A total of 25 triterpenoids, 13 flavonoids, 22 carbohydrates, 21 amino acids, 9 organic acid, 49 essential oils, and 14 heterocyclics within KF have been reported. Momordin Ic is a main substance, and it is usually used as a phytochemical marker for the quality control of KF. The pharmacological effects were achieved by the chemical constituents within KF. Hence, the interrelationship between compounds and pharmacological activities should be further studied. The pharmacokinetics of KF was lack, and a range of pharmacokinetic studies on its active compounds are needed to provide comprehensive data for clinical application. Altogether, this review extensively summarized phytochemistry, pharmacology, toxicity, quality control, and pharmacokinetic studies on KF to provide information for its further research and clinical applications.

## Figures and Tables

**Table 1:** Chemical compounds identified in Kochiae Fructus.

No.	Compounds	Formula	Ref.
Triterpenoids			
1	20-Hydroxyecdysone	C_27_H_44_O_7_	[5]
2	*β*-Sitosterol	C_29_H_50_O	[6]
3	Momordin Ic	C_41_H_64_O_13_	[7]
4	Oleanic-acid-3-*O*-*β*-D-xylopyranosly(1⟶3)-*β*-D-glucopyranoside	C_41_H_65_O_12_	[7]
5	Momordin I	C_41_H_64_O_13_	[7]
6	Oleanolic acid	C_30_H_48_O_3_	[7]
7	Oleanolic acid 3-*O*-glucuronide	C_36_H_56_O_9_	[7]
8	Oleanolic acid 3-*O*-*β*-D-glucopyranoside	C_36_H_56_O_8_	[7]
9	28-*O*-Deglucosyl-chikusetsusaponin V	C_42_H_66_O_14_	[7]
10	2′-*O*-Glucopyranosyl-momordin Ic	C_48_H_76_O_19_	[7]
11	Chikusetsusaponin V	C_48_H_76_O_19_	[7]
12	Momordin IIc	C_47_H_74_O_18_	[7]
13	Kochianoside I	C_36_H_56_O_10_	[8]
14	Kochianoside II	C_47_H_74_O_18_	[8]
15	Kochianoside III	C_41_H_64_O_14_	[8]
16	Kochianoside IV	C_41_H_64_O_13_	[8]
17	Daucosterol	C_35_H_60_O_6_	[6]
18	Oleanolic acid 3-*O*-*β*-D-xylopyranosyl(1⟶3)-*β*-D-glucopyranosiduronic acid 6-methyl ester	C_42_H_66_O_13_	[6]
19	3-*O*-*β*-D-Glucuronopyranosy-28-*O*-*β*-D-glucuronopyranosyl-oleanic-acid	C_42_H_66_O_14_	[6]
20	Oleanolic acid 3-*β*-D-glucopyranosiduronic acid 6-methyl ester	C_37_H_58_O_9_	[9]
21	Stigmasterol-3-*O*-*β*-D-glucopyranoside	C_35_H_58_O_6_	[8]
22	Oleanic-acid-3-*O*-[*β*-D-glucopyranosyl(1⟶2)-*β*-D-xylopyranosly(1⟶3)]-*β*-D-glucopyranosiduronic acid	C_47_H_74_O_18_	[10]
23	6′-Methyl ester of momordin Ic	C_42_H_66_O_13_	[11]
24	2′-*O*-*β*-D-Glucopyranosyl momordin Ic	C_47_H_74_O_18_	[11]
25	2′-*O*-*β*-D-Glucopyranosyl momordin IIc	C_53_H_84_O_23_	[11]

Flavonoids			
26	Quercetin 3-*O*-*β*-D-apiofuranosyl-(1⟶2)-*β*-D-galactopyranosyl-7-*O*-*β*-D-glucopyranoside	C_32_H_38_O_21_	[12]
27	Quercetin 3-*O*-*α*-L-rhamnopyranosyl-(1⟶6)-*β*-D-galactopyranosyl-7-*O*- *β*-D-sophoroside	C_39_H_50_O_26_	[12]
28	Quercetin 7-*O*-*β*-D-glucopyranoside	C_21_H_20_O_12_	[12]
29	Quercetin 3-*O*-*β*-D-sophoroside	C_27_H_30_O_17_	[12]
30	Quercetin 3-*O*-*β*-D-galactopyranosyl-7-*O*-*β*-D-glucopyranoside	C_27_H_30_O_17_	[12]
31	Quercetin 3-*O*-*β*-D-apiofuranosyl-(1⟶2)- *β*-D-galactopyranoside	C_26_H_28_O_16_	[12]
32	Isorhamnetin	C_16_H_12_O_7_	[6]
33	Quercetin	C_15_H_10_O_7_	[6]
34	Rutin	C_27_H_30_O_16_	[6]
35	Isorhamnetin-3-*O*-glucoside	C_22_H_22_O_12_	[6]
36	5,7,4′-Trihydroxy-6,3′-dimethoxyflavone	C_17_H_14_O_7_	[6]
37	5,7,4′-Dihydroxy-6-methoxyflavone	C_16_H_12_O_6_	[6]
38	Hyperoside	C_21_H_20_O_12_	[10]

Carbohydrates			
39	Sorbitol	C_6_H_14_O_6_	[13]
40	Glucose	C_6_H_12_O_6_	[13]
41	Galactopyranose	C_6_H_12_O_6_	[13]
42	Fructose	C_6_H_12_O_6_	[13]
43	Inositol-scyllo		[13]
44	Trehalose	C_12_H_22_O_11_	[13]
45	Sucrose	C_12_H_22_O_11_	[13]
46	*N*-Acetyl glucosamine	C_8_H_15_NO_6_	[13]
47	Mannitol	C_6_H_14_O_6_	[13]
48	Mannose	C_6_H_12_O_6_	[13]
49	Glucosamine	C_6_H_13_NO_5_	[13]
50	Glycerophosphoric acid	C_3_H_9_O_6_P	[13]
51	Rhamnose	C_6_H_12_O_5_	[13]
52	Ribitol	C_5_H_12_O_5_	[13]
53	Xylulose	C_5_H_10_O_5_	[13]
54	Erythritol	C_4_H_10_O_4_	[13]
55	2-*O*-Glycerol-*α*-D-galactopyranoside	C_9_H_18_O_8_	[13]
56	Maltitol	C_12_H_24_O_11_	[13]
57	Melibiose	C_12_H_22_O_11_	[13]
58	Xylose	C_5_H_10_O_5_	[13]
59	Ribose	C_5_H_10_O_5_	[13]
60	Arabinose	C_5_H_10_O_5_	[13]

Amino acids			
61	Tryptophan	C_11_H_12_N_2_O_2_	[13]
62	Tyrosine	C_9_H_11_NO_3_	[13]
63	Phenylalanine	C_9_H_11_NO_2_	[13]
64	Methionine	C_9_H_11_NO_2_	[13]
65	Glutamic acid	C_5_H_9_NO_4_	[13]
66	Lysine	C_6_H_14_N_2_O_2_	[13]
67	Glutamine	C_5_H_10_N_2_O_3_	[13]
68	Aspartic acid	C_4_H_7_NO_4_	[13]
69	Asparagine	C_4_H_8_N_2_O_3_	[13]
70	Leucine	C_6_H_13_NO_2_	[13]
71	Isoleucine	C_6_H_13_NO_2_	[13]
72	Pyroglutamic acid	C_5_H_7_NO_3_	[13]
73	Threonine	C_4_H_9_NO_3_	[13]
74	Valine	C_5_H_11_NO_2_	[13]
75	Proline	C_5_H_9_NO_2_	[13]
76	Serine	C_3_H_7_NO_3_	[13]
77	Alanine	C_3_H_7_NO_2_	[13]
78	Glycine	C_2_H_5_NO_2_	[13]
79	Ornithine	C_5_H_12_N_2_O_2_	[13]
80	Cysteine	C_3_H_7_NO_2_S	[13]
81	*β*-Alanine	C_3_H_7_NO_2_	[13]

Organic acid			
82	Glucaric acid	C_6_H_10_O_8_	[13]
83	Gluconic acid	C_6_H_12_O_7_	[13]
84	Glucuronic acid	C_6_H_10_O_7_	[13]
85	Citric acid	C_6_H_8_O_7_	[13]
86	Gluconic acid-1,5-lactone	C_6_H_10_O_6_	[13]
87	Threonic acid	C_4_H_8_O_5_	[13]
88	Malic acid	C_4_H_6_O_5_	[13]
89	Malonic acid	C_3_H_4_O_4_	[13]
90	Oxalic acid	C_2_H_2_O_4_	[13]

Essential oils			
91	*α*-Pinene	C_10_H_16_	[14]
92	2-Camphene	C_10_H_16_	[14]
93	9-Hexadecenoic acid	C_16_H_30_O_2_	[14]
94	Hexadecenoic acid	C_16_H_30_O_2_	[14]
95	Ethyl hexadecenoic acid	C_18_H_34_O_2_	[14]
96	9,12-Octadecadienoic acid	C_18_H_32_O_2_	[14]
97	Ethyl 9,12-octadecadienoic acid	C_20_H_36_O_2_	[14]
98	9-Ethyl octadecanoate	C_20_H_38_O_2_	[14]
99	Stearic acid	C_18_H_36_O_2_	[14]
100	Methyl hexadecanoate	C_17_H_34_O_2_	[14]
101	Methyl hexadecenoic acid	C_17_H_32_O_2_	[14]
102	Octadeca-9,12,15-trienoic acid	C_18_H_30_O_2_	[14]
103	Methyl 9-octadecenoic acid	C_19_H_36_O_2_	[14]
104	Methyl 10-octadecenoic acid	C_19_H_36_O_2_	[14]
105	Octadecenoic acid	C_19_H_34_O_2_	[14]
106	Methyl 11-eicosenoic acid	C_21_H_40_O_2_	[14]
107	Methyl eicosenoic acid	C_21_H_42_O_2_	[14]
108	Benzene, 1-methyl-2-propyl-	C_10_H_14_	[15]
109	Cyclohexene, 1-methyl-4-(1-methylethenyl)-	C_10_H_10_	[15]
110	(E)-3,7-Dimethyl-2,6-octadienal	C_10_H_16_O	[15]
111	Methyl 2-methyl-octanate	C_10_H_20_O_2_	[15]
112	Benzene, 1-methoxy-4-(1-propenyl)-	C_10_H_12_O	[15]
113	Methyl nonanoate	C_10_H_20_O	[15]
114	1-Undecyne	C_11_H_20_	[15]
115	Ethyl nonanoate	C_11_H_22_O_2_	[15]
116	Naphthalene	C_11_H_10_	[15]
117	Bicyclo(4,3,1)decan-10-one	C_10_H_16_O	[15]
118	5-Ethyl-2-nonanol	C_11_H_24_O	[15]
119	4,8-Dimethyl-1-nonanol	C_11_H_24_O	[15]
120	(Z)-*β*-Farnesene	C_15_H_24_	[15]
121	*trans*-*β*-Farnesene	C_15_H_24_	[15]
122	2,6-Di-T-butyl-1,4-benzoquinone	C_14_H_20_O	[15]
123	*β*-Ionone	C_13_H_20_O	[15]
124	Naphthalene, 1,2,3,5,6,7,8a-octahydro-1,8a-demethyl-7-(1-methyethyl)-[1R-(1*α*,7*β*,8a*α*)]-	C_15_H_24_	[15]
125	*β*-Chamigrene	C_15_H_24_	[15]
126	Ethyl dodecanoate	C_14_H_28_O_2_	[15]
127	Hexadecane	C_16_H_34_	[15]
128	Methyl tetradecanoate	C_15_H_30_O_2_	[15]
129	Benzene, 1,1'-(1,2-ethynediyl)bis-	C_14_H_10_	[15]
130	Octadecane	C_18_H_38_	[15]
131	2-Pentadecanone, 6,10,14-trimethyl-	C_18_H_36_O	[15]
132	Ethyl pentadecanoate	C_17_H_34_O_2_	[15]
133	Ethyl hexadecanoate	C_18_H_36_O_2_	[15]
134	Eicosane	C_20_H_42_	[15]
135	Methyl octadecadienoate	C_19_H_34_O_2_	[15]
136	Heneicosane	C_21_H_44_	[15]
137	Ethyl-octadecadienoate	C_20_H_36_O_2_	[15]
138	Ethyl octadecanoate	C_20_H_40_O_2_	[15]
139	Docosane	C_22_H_46_	[15]

Heterocyclics			
140	Oleamide	C_18_H_35_NO	[13]
141	Ethanolamine	C_2_H_7_NO	[13]
142	Uridine	C_9_H_12_N_2_O_6_	[13]
143	Uric acid	C_5_H_4_N_4_O_3_	[13]
144	Guanine	C_5_H_5_N_5_O	[13]
145	Adenine	C_5_H_5_N_5_	[13]
146	Spermidine	C_7_H_19_N_3_	[13]
147	Putrescine	C_4_H_12_N_2_	[13]
148	Ethylene glycol	C_2_H_6_O_2_	[13]
149	Urea	CH_4_N_2_O	[13]
150	Allantoin	C_4_H_6_N_4_O_3_	[13]
151	Thymine	C_5_H_6_N_2_O_2_	[13]
152	2-(4-Hydroxy-3-methoxyphenyl)-ethanol	C_9_H_12_O_3_	[13]
153	Dopamine	C_8_H_11_NO_2_	[16]

**Table 2:** Traditional uses of Kochiae Fructus (KF) referring to database “https://www.yaozh.com.”

Traditional uses	Reference
KF is used to treat frequent urination, urinary incontinence, and abnormal leucorrhea and has an effect of strengthening ears	Ben Cao Qiu Yuan
KF is used to treat frequent urination and urinary incontinence and has an effect of strengthening ears	Ben Cao Qiu Zhen
KF has an effect of heat-clearing	Ben Cao Shu
KF is used to treat skin itching and eczema and has effects of reducing swelling and improving eyesight and ears	Yao Jian
KF is good for urinating and has effects of improving eyesight and ears and antiaging	Ben Jing
KF is used to treat eczema	Bie Lu
KF has an effect for urinating	Ben Cao Zheng Yi
KF is used to treat male impotence	Yao Xing Lun
KF has an effect of reducing swelling	Ri Hua Zi Ben Cao
KF is used to improve urination problems, treat rubella, and abnormal vaginal discharge	Dian Nan Ben Cao
KF is used to treat itchy skin	Ben Cao Yuan Shi
KF can help urination	Ben Cao Bei Yao
KF is used to treat swelling and pain of the head and eyes, back pain, blood in the stool, and malignant sores	Yu Qiu Yao Jie

**Table 3:** Pharmacological effects of Kochiae Fructus (KF).

Pharmacological effect	Model	Administration	Minimal active concentration	Reference
Anti-inflammatory effect	The ddY mice in an acetic acid-induced vascular permeability	Tested drug: 70% ethanol extract of KF at the doses of 50, 200, and 500 mg/kg, p.o. Positive control: indomethacin at 10 mg/kg	200 mg/kg	[19]
	The ddY mice in a carrageenin-induced edema	Tested drug: 70% ethanol extract of KF at the doses of 50, 200, and 500 mg/kg, p.o. Positive control: indomethacin at 10 mg/kg	200 mg/kg	[19]
	The ddY mice in a compound 48/80-induced edema	Tested drug: 70% ethanol extract of KF at the doses of 50, 200, and 500 mg/kg, p.o. Positive control: diphenhydramine at 50 mg/kg	500 mg/kg	[19]
	The ddY mice in a chemical mediator-induced edema	Tested drug: 70% ethanol extract of KF at the doses of 50, 200, and 500 mg/kg, p.o. Positive control: cyproheptadine at 2 mg/kg; diphenhydramine at 50 mg/kg	200 mg/kg	[19]
	The isolated ileum of guinea pig in a histamine-induced contraction	Tested drug: 70% ethanol extract of KF at the doses of 10, 50, 100, and 300 *μ*g/mL	220 *μ*g/mL	[19]
	The ddY mice in an arachidonic acid-induced edema	Tested drug: 70% ethanol extract of KF at the doses of 50, 200, and 500 mg/kg, p.o. Positive control: phenidone at 20 mg/kg, i.v.	500 mg/kg	[19]
	A picryl chloride-induced ear inflammatory model in ICR mice	Tested drug: 70% ethanol extract of KF at the doses of 100, 200, and 500 mg/kg, i.g. Positive control: prednisone at 50 mg/kg	500 mg/kg	[20]
	A dinitrochlorobenzene-induced allergic contact dermatitis model in rats	Tested drug: total flavonoids of KF at the doses of 100 and 200 mg/kg. p.o. Positive control: sodium prednisolone acetate at 2.5 *μ*g/mL	100 mg/kg	[21]
	A DNFB-induced contact dermatitis model in mice	Tested drug: methanol extract of KF at the doses of 30, 100, and 300 *μ*g/ear for external use Positive control: dexamethasone at 75 *μ*g/ear	100 *μ*g/ear	[22]
	The ddY mice in a carrageenin-induced edema	Tested drug: momordin Ic at the doses of 20, 50, and 100 mg/kg, p.o. Positive control: indomethacin at 10 mg/kg	20 mg/kg	[19]
	LPS-stimulated RAW 264.7 cell line	Tested drug: 20-hydroxyecdysone, momordin Ic, and oleanolic acid with various concentrations (6.25, 12.5, or 25 *μ*M) Positive control: indomethacin at 2.5 ng/mL	6.25 *μ*M	[5]
Hypoglycemic effect				
	Normal, alloxan-induced hyperglycemic and insulin-induced hypoglycemic mice	Tested drug: *n*-butanol extract of KF at the doses of 25 and 50 mg/kg, p.o.	25 mg/kg	[23]
	Examining the activity of *α*-glucosidase in rat intestine in vitro	Tested drug: *n*-butanol extract of KF at the doses of 62.5, 125, 250, and 500 *μ*g/mL Positive control: acarbose at 2 *μ*g/mL	125 *μ*g/mL	[23]
	Examining the ability of glucose absorption in rat intestine in vitro	Tested drug: *n*-butanol extract of KF at the doses of 0, 100, 200, 400, and 800 *μ*g/mL	100 *μ*g/mL	[23]
	Testing the propulsive function of small intestine in normal, fenfluramine-treated, dopamine-treated, acetic acid-treated, and N*ω*-nitro-L-arginine-treated rats	Tested drug: *n*-butanol extract of KF at the doses of 25 and 50 mg/kg, p.o.	50 mg/kg	[24]
	Testing the gastric emptying in rats	Tested drug: oleanolic acid 3-*O*-glucuronide and momordin Ic at the doses of 12.5, 25, and 50 mg/kg, p.o. Positive control: atropine sulfate at 10 mg/kg	25 and 12.5 mg/kg, respectively	[25]
	Testing the glucose uptake in rat small intestine in vitro	Tested drug: oleanolic acid 3-O-glucuronide and momordin Ic at the doses of 0, 5, 50, and 500 *μ*M Positive control: phlorizin at 1 *μ*M	50 and 5 *μ*M, respectively	[25]
	Gastric emptying test on 1.5% carboxymethyl cellulose sodium salt test meal-loaded mice, 40% glucose test meal-loaded mice, milk test meal-loaded mice, and 60% ethanol test meal-loaded mice	Tested drug: momordin Ic in the dose range of 12.5–50 mg/kg, p.o.	50 mg/kg	[3]

Anticancer effects				
Antiliver cancer	Inducing apoptosis of human hepatocyte carcinoma HepG2 cells	Tested drug: momordin Ic in the concentration range of 10–30 *μ*M	15 *μ*M	[26]
	Inducing autophagy of human hepatocyte carcinoma HepG2 cells	Tested drug: momordin Ic in the dose range of 5–20 *μ*M	10 *μ*M	[27]
	Inducing apoptosis of human hepatocyte carcinoma HepG2 cells	Tested drug: momordin Ic in the dose range of 5, 10, and 15 *μ*M	5 *μ*M	[28]
	Suppressing invasion of human hepatocyte carcinoma HepG2 cells	Tested drug: momordin Ic at the dose of 10 *μ*M	10 *μ*M	[29]
	Inhibiting migration and invasion of human hepatocyte carcinoma HepG2 cells	Tested drug: momordin Ic in the dose range of 1–10 *μ*M	5 *μ*M	[30]

Antiprostate cancer	VEGF-induced angiogenesis in human umbilical vein endothelial cells and proliferation in prostate cancer cells	Tested drug: methanol extract of *KF* in the dose range of 10–20 *μ*g/mL and 10–250 *μ*g/mL, respectively	20 *μ*g/mL and 100 *μ*g/mL, respectively	[31]
	Testing the SUMO-specific protease 1 in prostate cancer cells and a xenograft PC3 tumor mouse model	Tested drug: momordin Ic at the dose of 6.25, 12.5, and 25 *μ*M and 10 mg/kg/day, i.p. For 20 days, respectively	IC_50_ was 15.37 *μ*M and 10 mg/kg/day	[32]
Antifungal effect				
	*In vitro* for *Trichophyton mentagrophytes*, *Trichophyton rubrum*, *Microsporum canis*, *Trichophyton violaceum*, and *Trichophyton schoenleinii*	Tested drug: water extract of KF in the concentration range of 0.04%–25%	3.12%	[33]
	Inhibiting the growing of *Fusarium graminearum, Fusarium oxysporum, Monilia cinerea, Physalos porapiricola, Alternaria alternata,* and *Valsa mali*	Tested drug: water, petroleum ether, chloroform, ethylacetate, and methanol extract of *KF* with the concentration of dose 1 mg/15 mL	1 mg/15 mL	[34]
	*In vitro* for *Microsporum ferrugineum, Microsporum gypseum, Trichophyton schoenleini, Trichophyton mentagrophytes, Trichophyton violaceum, Trichophyton rubrum, Epidermophyton floccosum, Aspergillus fumigatus, Candida albicans, Cryptococcus neoformans*	Tested drug: the saponin extract, flavone extract I (40% alcohol eluent), flavone extract II (80% alcohol eluent), and lipid extract with no concentration mentioned	All samples have the antifungal effect on *Microsporum ferrugineum and Trichophyton rubrum*, but the concentration was not mentioned	[35]

Antipruritogenic effect				
	A compound 48/80-induced pruritogenic model in male ddY mice	Tested drug: 70% ethanol extract of KF at the dose of 200 and 500 mg/kg, p.o.	200 mg/kg	[36]
		Tested drug: methanol extract of KF at the dose of 50, 200, and 500 mg/kg, p.o.	500 mg/kg	
		Tested drug: momordin Ic at the dose of 20, 50, and 100 mg/kg, p.o. Positive control: diphenhydramine at 20 mg/kg	50 mg/kg	
	Itching guinea pig model caused by histamine itching mice model	Tested drug: water extract of KF at the concentration of 0.15 g/mL, 0.3 g/mL, and 0.6 g/mL for external use Positive control: cyproheptadine	0.15 g/mL	[33]

Antinociceptive effect				
	The ddY mice in an acetic acid-induced writhing test	Tested drug: 70% ethanol extract of KF at the dose of 50, 200, and 500 mg/kg, p.o.	500 mg/kg	[19]
		Tested drug: momordin Ic at the dose of 20, 50, and 100 mg/kg, p.o. Positive control: aspirin at 200 mg/kg	20 mg/kg	

Inhibition effect on hypersensitivity				
	DTH models upon challenge with SRBC or PC	Tested drug: 70% ethanol extract of KF at the dose of 100, 200, and 500 mg/kg. Positive control: prednisone at 50 mg/kg	100 mg/kg	[20]
		Tested drug: total saponins extract of KF at the dose of 50, 100, and 200 *μ*g/mL Positive control: cyproheptadine at 20 mg/kg	200 mg/kg	

Myocardial protection				
	A furazolidone-induced dilated cardiomyopathy model in Wistar rats	Tested drug: water extract of KF at the dose of 5 and 20 mg/g, p.o. Negative model: water	20 mg/g	[37]
Hepatoprotective effect				
	Carbon tetrachloride-induced liver damage in rats	Tested drug: momordin Ic and oleanolic acid at the dose of 30 mg/kg/day for 14 days, p.o. Negative control: saline	30 mg/kg/day for 14 days	[38]

Protecting gastric mucosal lesions				
	Ethanol-induced gastric mucosal lesions in rats and indomethacin-induced gastric mucosal lesions in rats	Tested drug: momordin Ic in the dose range of 2.5–50 mg/kg, p.o. Positive control: omeprazole at 20 or 5 mg/kg, respectively	5 mg/kg	[39]

Suppressing osteoclastogenesis				
	A cocultured system and a RANKL-induced osteoclast precursor system	Tested drug: momordin Ic in the dose range of 0.1–5 *μ*M	0.5 *μ*M	[40]

Antibacteria effect				
	Minimum inhibitory concentration (MIC) test on *Escherichia coli*	Tested drug: oleanolic acid in the dose range of 15.6–4000 *μ*g/mL	31.3 *μ*g/mL	[41]

## Data Availability

The data used to support the findings of this study are available from the corresponding author upon request.
